# The relationship between dietary index for gut microbiota and hyperuricemia: a cross-sectional study using NHANES data

**DOI:** 10.3389/fnut.2025.1580122

**Published:** 2025-05-26

**Authors:** Xin Cai, Shaoqin Zhang, Tianzuo Lan, Zexu Jin, Jiajun Liu, Zong Jiang, Qingwan Yang

**Affiliations:** ^1^The First People's Hospital of Guiyang, Guiyang, China; ^2^The Second Clinical Medical College of Guizhou University of Traditional Chinese Medicine, Guiyang, China

**Keywords:** hyperuricemia, dietary index for gut microbiota, diet index, nutritional epidemiology, NHANES

## Abstract

**Background:**

Hyperuricemia (HUA) is a common metabolic disorder, yet the impact of diet and gut microbiota on uric acid metabolism remains insufficiently understood. This study aims to investigate the relationship between the dietary index for gut microbiota (DI-GM) and HUA using data from the National Health and Nutrition Examination Survey (NHANES).

**Methods:**

This study employed NHANES data gathered from 2007 to 2020, encompassing 25,899 adults aged 18 years and older. The DI-GM, which spans from 0 to 14, was calculated based on dietary recall information. The diagnosis of HUA was established through laboratory findings. To examine the relationship between DI-GM and HUA, multivariable logistic regression was utilized, accounting for pertinent confounding variables.

**Results:**

Upon adjusting for possible confounding variables, an elevated DI-GM score demonstrated a notable correlation with a reduced likelihood of HUA [odds ratio (OR) = 0.968, 95% confidence interval (CI) = 0.949–0.987, *p* = 0.005]. Subjects exhibiting a DI-GM score of ≥6 demonstrated a markedly reduced risk of HUA in contrast to those scoring between 0 and 3 (OR = 0.897, 95% CI = 0.821–0.980, *p* = 0.016).

**Conclusion:**

A higher DI-GM score is inversely related to the risk of HUA.

## 1 Introduction

Hyperuricemia (HUA) is a metabolic disorder that manifests through disruptions in purine metabolism, leading to increased serum uric acid (UA) concentrations. The worldwide occurrence of HUA has been on the rise, primarily influenced by shifts in lifestyle and dietary habits. From 1990 to 2021, the global incidence of gout escalated from 93.097 to 109.075 per 100,000 individuals, respectively (1). In China, the prevalence of HUA among males is noted to be 21.5% (2). HUA is not only the principal factor contributing to gout but is also linked to chronic kidney disease (CKD) (3), cardiovascular adverse events (4), and metabolic syndrome (5), highlighting its significance as a public health issue on a global scale.

Growing evidence highlights the important role of diet in the development and management of HUA (6). Meta-analyses and population-based studies have demonstrated that high consumption of red meat, seafood, alcohol, and fructose is positively associated with elevated serum UA levels and an increased risk of HUA (7–9). In contrast, intake of dairy products, soy foods, vegetables, and coffee has been linked to lower UA concentrations (7). Additionally, recent studies have reported that pro-inflammatory diets and ultra-processed food consumption may adversely affect UA metabolism (10, 11), whereas healthier dietary patterns, such as the Dietary Approaches to Stop Hypertension and Mediterranean diets, are associated with reduced serum UA levels and a lower likelihood of HUA (12–14). These findings support a shift from nutrient-specific evaluations toward holistic assessments of overall dietary patterns. Moreover, emerging evidence suggests that some of the health effects of diet may be mediated through its influence on the gut microbiota (15), underscoring the potential value of incorporating microbiota-related dietary indices into future HUA research.

Recent advances in nutritional science have increasingly highlighted the gut microbiota as a key mediator of dietary effects on human health (16). Accumulating evidence suggests that long-term dietary patterns play a critical role in shaping the structure and function of the gut microbiome (17). Diets rich in fiber, polyphenols, and unsaturated fats are associated with greater microbial diversity and increased abundance of beneficial taxa, whereas high-fat, high-sugar, and ultra-processed diets are linked to dysbiosis and metabolic disturbances (15, 18). Emerging research also indicates that alterations in gut microbiota may influence UA metabolism through pathways involving purine degradation, microbial metabolite production, and UA excretion (19–22). In this context, the dietary index for gut microbiota (DI-GM) has been developed to assess the microbiota-related quality of diet by incorporating components known to affect gut microbial composition (23). DI-GM has shown consistent inverse associations with several chronic conditions, including stroke, diabetes, and fatty liver disease (24–26). However, its association with HUA has not yet been investigated. Given the increasing interest in microbiota-oriented dietary strategies, exploring this relationship may offer epidemiological insights into the dietary determinants of HUA.

This study aimed to investigate whether the DI-GM is independently associated with HUA, based on data from the National Health and Nutrition Examination Survey (NHANES). The findings may help clarify the potential role of microbiota-related dietary patterns in the development or management of HUA.

## 2 Methods

### 2.1 Data source

The NHANES dataset, which spans 2007–2020 and is accessible to the public, was the source of the data in this investigation. The NHANES is a cross-sectional study that uses a multi-stage probability sampling method to evaluate the nutritional and health status of the American people. A participants gave their written informed consent.

**Figure 1:**
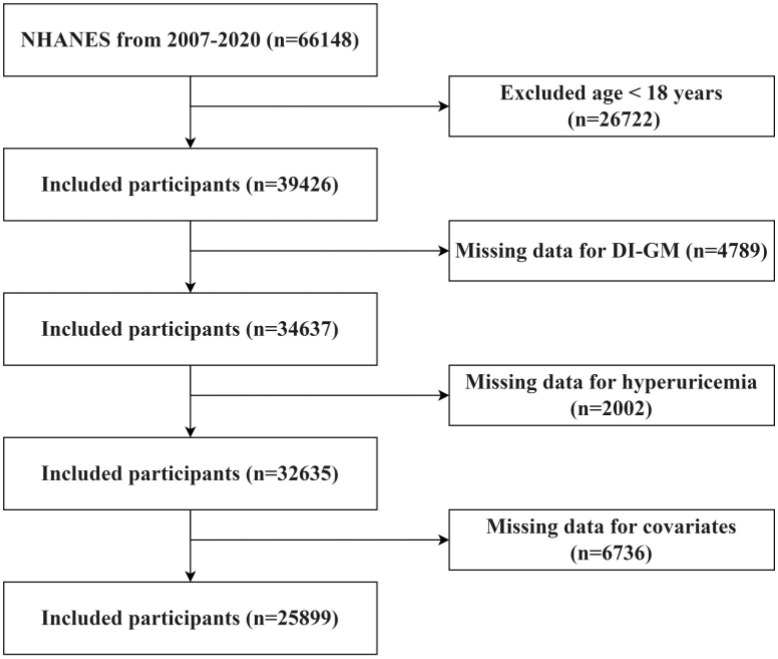
Flowchart of participant screening in NHANES from 2007 to 2020.

The research concentrated on individuals who are 18 years of age and above. Individuals who fulfilled any of the subsequent criteria were omitted from the study: (1) persons younger than 18 years of age (*n* = 26,722); (2) participants with incomplete DI-GM data (*n* = 4,789); (3) individuals with missing UA levels (*n* = 2,002); and (4) those without covariate data (*n* = 6,736). The process for selecting participants is depicted in Figure 1.

### 2.2 Study subjects

The study subjects were adults aged 18 years or older who had completed dietary surveys and UA tests. Exclusion criteria were individuals with missing or incomplete data.

### 2.3 DI-GM scoring criteria

Based on the DI-GM scoring criteria (23), the impact of 14 foods or nutrients on the GM was evaluated, including dietary fiber, polyphenols, high-fat foods, and processed meats. A score of 1 was assigned for positively scored foods if the daily intake exceeded the recommended amount, and a score of 0 was assigned otherwise. The total DI-GM score ranged from 0 to 14, with higher scores indicating a more beneficial impact of the diet on the GM.

### 2.4 Definition of HUA

HUA was diagnosed in males as a fasting serum UA level >7.0 mg/dl (420 μmol/L) and in females as >6.0 mg/dl (360 μmol/L) (27, 28).

### 2.5 Covariates

Covariates considered in this study, based on existing literature, included: (1) demographic factors such as age and poverty-income ratio (PIR); (2) lifestyle factors like smoking, alcohol consumption, and BMI; and (3) comorbid conditions, including hypertension, diabetes, hyperlipidemia, and CKD.

### 2.6 Statistical analysis

Participants were classified into four distinct quartiles according to their DI-GM scores: Q1 (0–3), Q2 (4), Q3 (5), and Q4 (≥6). Data were expressed as means ± standard deviations (SD). Comparative analyses of quartiles were performed utilizing analysis of variance (ANOVA) or the Kruskal-Wallis test for continuous variables, alongside the chi-square test for categorical variables. Logistic regression analysis was utilized to evaluate the relationship between DI-GM and HUA, employing odds ratio (OR) and 95% CI to quantify the association. Three distinct models underwent evaluation: Model 1, which remained unadjusted; Model 2, which incorporated adjustments for age, gender, race, education level, marital status, and poverty income ratio (PIR); and Model 3, which included adjustments for smoking, alcohol consumption, body mass index, hypertension, diabetes, hyperlipidemia, and CKD, alongside the variables accounted for in Model 2. The sensitivity analyses encompassed subgroup analysis, interaction tests, and restricted cubic spline (RCS) analysis. All statistical analyses were conducted utilizing R version 4.3.3, establishing a significance threshold at *p* < 0.05.

## 3 Results

### 3.1 Basic characteristics of the study population

A total of 66,148 participants were initially registered between 2007 and 2010, with 25,899 individuals matching the inclusion criteria for the final study sample. Participants were sorted into four DI-GM score quartiles. Significant differences were seen in age, gender, education level, marital status, PIR, smoking, alcohol intake, BMI, and diabetes across the quartiles. The baseline characteristics of the individuals are summarized in Table 1.

**Table 1:** Basic characteristics of the participants.

**DI-GM quartile**	**Q1 (0–3)**	**Q2 (4)**	**Q3 (5)**	**Q4 (≥6)**	***p*-Value**
*N*	6,342	6,361	5,970	7,226	
Age (years)	47.40 (17.37)	47.73 (17.68)	49.41 (17.58)	51.71 (17.20)	< 0.001
**Sex (%)**					
Male	3,321 (52.37)	3,177 (49.94)	2,930 (49.08)	3,334 (46.14)	< 0.001
Female	3,021 (47.63)	3,184 (50.06)	3,040 (50.92)	3,892 (53.86)	
**Race (%)**					
Mexican American	875 (13.80)	1,042 (16.38)	921 (15.43)	896 (12.40)	< 0.001
Other Hispanic	686 (10.82)	681 (10.71)	591 (9.90)	652 (9.02)	
Non-Hispanic white	2,524 (39.80)	2,594 (40.78)	2,634 (44.12)	3,607 (49.92)	
Non-Hispanic black	1,681 (26.51)	1,407 (22.12)	1,178 (19.73)	1,111 (15.38)	
Other race	576 (9.08)	637 (10.01)	646 (10.82)	960 (13.29)	
**Education (%)**					
Less than high school	1,549 (24.42)	1,638 (25.75)	1,268 (21.24)	1,221 (16.90)	< 0.001
High school or equivalent	1,742 (27.47)	1,494 (23.49)	1,345 (22.53)	1,327 (18.36)	
College or above	3,051 (48.11)	3,229 (50.76)	3,357 (56.23)	4,678 (64.74)	
**Marital status (%)**					
Married and a partner	3,637 (57.35)	3,652 (57.41)	3,636 (60.90)	4,522 (62.58)	< 0.001
Never married	1,351 (21.30)	1,291 (20.30)	1,083 (18.14)	1,080 (14.95)	
Widowed, divorced or separated	1,354 (21.35)	1,418 (22.29)	1,251 (20.95)	1,624 (22.47)	
**Poverty to income ratio (%)**					
< 1.3	2,244 (35.38)	2,203 (34.63)	1,815 (30.40)	1,734 (24.00)	< 0.001
1.3–3.5	2,537 (40.00)	2,403 (37.78)	2,180 (36.52)	2,577 (35.66)	
>3.5	1,561 (24.61)	1,755 (27.59)	1,975 (33.08)	2,915 (40.34)	
**Smoking (%)**					
Never smoker	3,361 (53.00)	3,519 (55.32)	3,337 (55.90)	4,133 (57.20)	< 0.001
Current smoker	1,527 (24.08)	1,376 (21.63)	1,208 (20.23)	1,131 (15.65)	
Former smoker	1,454 (22.93)	1,466 (23.05)	1,425 (23.87)	1,962 (27.15)	
**Drinking (%)**					
Never drinker	2,821 (44.48)	2,915 (45.83)	2,944 (49.31)	3,794 (52.50)	< 0.001
Current drinker	2,554 (40.27)	2,538 (39.90)	2,183 (36.57)	2,383 (32.98)	
Former drinker	967 (15.25)	908 (14.27)	843 (14.12)	1,049 (14.52)	
**Body mass index (%)**					
Under weight (< 18.5)	89 (1.40)	103 (1.62)	97 (1.62)	81 (1.12)	< 0.001
Normal (18.5 to < 25)	1,497 (23.60)	1,620 (25.47)	1,544 (25.86)	2,147 (29.71)	
Overweight (25 to < 30)	1,973 (31.11)	2,077 (32.65)	1,944 (32.56)	2,472 (34.21)	
Obese (30 or greater)	2,783 (43.88)	2,561 (40.26)	2,385 (39.95)	2,526 (34.96)	
**Hypertension (%)**					
No	3,570 (56.29)	3,707 (58.28)	3,451 (57.81)	4,203 (58.16)	0.083
Yes	2,772 (43.71)	2,654 (41.72)	2,519 (42.19)	3,023 (41.84)	
**Diabetes (%)**					
No	5,038 (79.44)	5,141 (80.82)	4,909 (82.23)	5,980 (82.76)	< 0.001
Yes	1,304 (20.56)	1,220 (19.18)	1,061 (17.77)	1,246 (17.24)	
**Hyperlipidemia (%)**					
No	1,805 (28.46)	1,829 (28.75)	1,735 (29.06)	2,074 (28.70)	0.908
Yes	4,537 (71.54)	4,532 (71.25)	4,235 (70.94)	5,152 (71.30)	
**CKD (%)**					
No	5,186 (81.77)	5,263 (82.74)	4,929 (82.56)	6,028 (83.42)	0.091
Yes	1,156 (18.23)	1,098 (17.26)	1,041 (17.44)	1,198 (16.58)	
**Hyperuricemia (%)**					
0	4,972 (78.40)	5,056 (79.48)	4,769 (79.88)	5,833 (80.72)	< 0.001
1	1,370 (21.60)	1,305 (20.52)	1,201 (20.12)	1,393 (19.28)	

### 3.2 Relationship between DI-GM scores and UA levels

**Figure 2:**
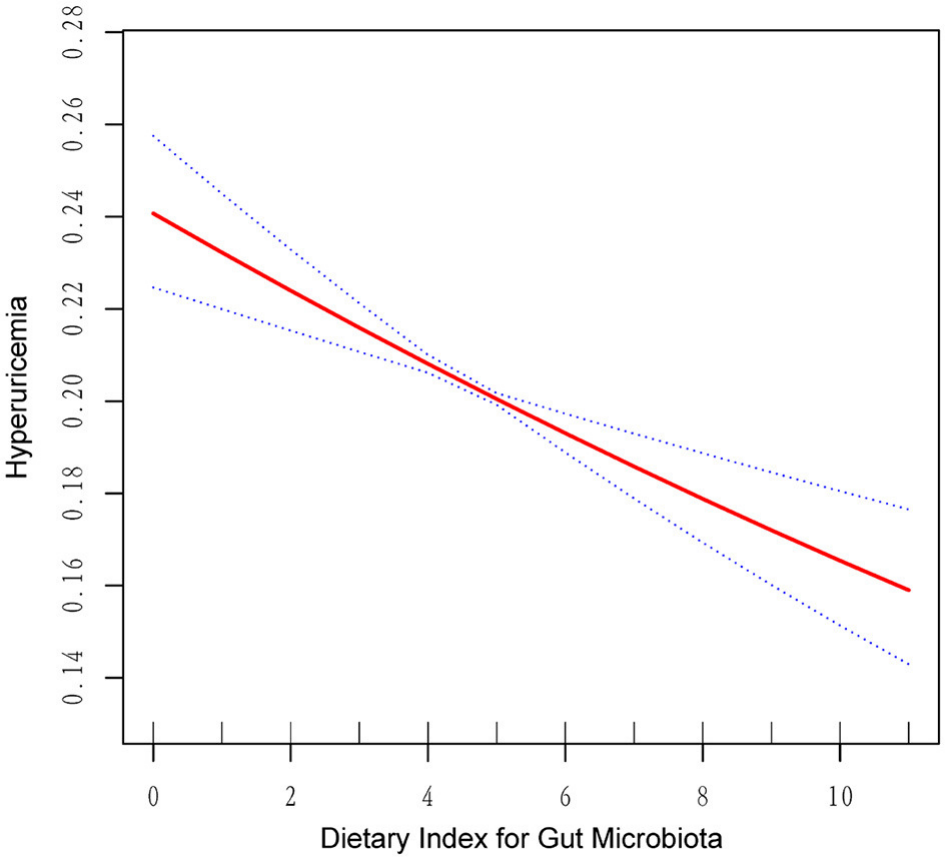
RCS plot of the association between DI-GM and HUA.

As shown in Table 2, multivariate logistic regression analysis revealed a significant inverse association between higher DI-GM scores and the likelihood of HUA. In the unadjusted model, each unit increase in the DI-GM score corresponded to a 3.9% reduction in the risk of HUA (OR = 0.961, 95% CI = 0.943–0.979, *p* < 0.001). The association strengthened further in Model I, which adjusted for confounding variables (OR = 0.954, 95% CI = 0.935–0.972, *p* < 0.001). In Model II, the negative correlation remained consistent (OR = 0.968, 95% CI = 0.949–0.987, *p* = 0.005). Notably, the risk of HUA was lower in the Q4 group (OR = 0.897, 95% CI = 0.821–0.980, *p* = 0.016). RCS analysis (Figure 2) further illustrated a linear negative relationship between DI-GM scores and HUA risk.

**Table 2:** Association between DI-GM and HUA by logistic regression.

**Exposure**	**Model 1**	**Model 2**	**Model 3**
	**OR (95% CI)** ***p-*****value**	**OR (95% CI)** ***p-*****value**	**OR (95% CI)** ***p-*****value**
DI-GM continuous	0.961 (0.943,0.979) < 0.001	0.954 (0.935,0.972) < 0.001	0.968 (0.949,0.987) 0.005
**DI-GM quartile**			
Q1 (0–3)	1	1	1
Q2 (4)	0.937 (0.860, 1.020) 0.133	0.955 (0.876, 1.042) 0.301	0.972 (0.890, 1.062) 0.533
Q3 (5)	0.914 (0.838, 0.997) 0.043	30.913 (0.836, 0.998) 0.045	0.957 (0.874, 1.048) 0.343
Q4 (≥6)	0.867 (0.797, 0.942) < 0.001	0.841 (0.771, 0.917) < 0.001	0.897 (0.821, 0.980) 0.016
*p* for trend	< 0.001	< 0.001	< 0.001

Model 1, which remained unadjusted; Model 2, which incorporated adjustments for age, gender, race, education level, marital status, and poverty income ratio (PIR); and Model 3, which included adjustments for smoking, alcohol consumption, body mass index, hypertension, diabetes, hyperlipidemia, and CKD, alongside the variables accounted for in Model 2.

In the fully adjusted model (Table 3), several components categorized as beneficial to gut microbiota demonstrated statistically significant inverse associations with hyperuricemia. Specifically, higher intakes of fiber (OR = 0.751, 95% CI = 0.705–0.801), whole grains (OR = 0.827, 95% CI = 0.770–0.889), fermented dairy (OR = 0.900, 95% CI = 0.843–0.961), avocado (OR = 0.786, 95% CI = 0.640–0.965), cranberry (OR = 0.838, 95% CI = 0.728–0.964), chickpea (OR = 0.611, 95% CI = 0.425–0.877), and coffee (OR = 0.920, 95% CI = 0.857–0.988) were all associated with a lower risk of hyperuricemia. Conversely, no significant associations were observed for soybean, broccoli, or green tea, nor for any components classified as unfavorable to gut microbiota.

**Table 3:** Relationship between DI-GM components and HUA.

**Components**	**Model 1**	**Model 2**	**Model 3**
	**OR (95% CI)** ***p-*****value**	**OR (95% CI)** ***p-*****value**	**OR (95% CI)** ***p-*****value**
**Beneficial to gut microbiota**			
Fermented dairy	0.818 (0.768, 0.871) < 0.001	0.911 (0.854, 0.972) 0.005	0.900 (0.843, 0.961) 0.002
Whole grains	0.888 (0.829, 0.951) 0.001	0.817 (0.761, 0.876) < 0.001	0.827 (0.770, 0.889) < 0.001
Fiber	0.728 (0.685, 0.773) < 0.001	0.746 (0.701, 0.794) < 0.001	0.751 (0.705, 0.801) < 0.001
Soybean	1.032 (0.949, 1.123) 0.456	1.046 (0.961, 1.140) 0.298	1.064 (0.976, 1.160) 0.160
Broccoli	0.937 (0.838, 1.048) 0.258	0.961 (0.857, 1.077) 0.492	0.973 (0.866, 1.092) 0.639
Avocado	0.644 (0.527, 0.786) < 0.001	0.766 (0.626, 0.938) 0.010	0.786 (0.640, 0.965) 0.021
Cranberry	0.828 (0.723, 0.948) 0.006	0.832 (0.725, 0.956) 0.009	0.838 (0.728, 0.964) 0.013
Chickpea	0.539 (0.378, 0.768) 0.001	0.590 (0.412, 0.845) 0.004	0.611 (0.425, 0.877) 0.008
Coffee	0.997 (0.934, 1.065) 0.933	0.911 (0.850, 0.976) 0.008	0.920 (0.857, 0.988) 0.022
Green tea	1.099 (1.008, 1.198) 0.033	1.089 (0.997, 1.189) 0.059	1.085 (0.992, 1.186) 0.074
**Unfavorable to gut microbiota**			
Red meat	1.009 (0.948, 1.073) 0.786	0.987 (0.926, 1.051) 0.677	1.008 (0.946, 1.075) 0.799
Processed meat	0.978 (0.912, 1.049) 0.532	1.015 (0.946, 1.090) 0.675	1.059 (0.985, 1.139) 0.118
Refined grains	0.957 (0.917, 0.999) 0.043	0.960 (0.918, 1.003) 0.069	0.973 (0.932, 1.016) 0.212
Fat	0.949 (0.886, 1.017) 0.137	0.979 (0.913, 1.050) 0.555	1.012 (0.942, 1.087) 0.743

Model 1, which remained unadjusted; Model 2, which incorporated adjustments for age, gender, race, education level, marital status, and poverty income ratio (PIR); and Model 3, which included adjustments for smoking, alcohol consumption, body mass index, hypertension, diabetes, hyperlipidemia, and CKD, alongside the variables accounted for in Model 2.

### 3.3 Subgroup analysis and interaction test between DI-GM and HUA

Participants were stratified by various characteristics. As shown in Table 4, the relationship between DI-GM and HUA differed significantly across categories, indicating that factors such as age (excluding 40–59 years), education level (college or above), marital status (excluding Never married), PIR (excluding < 1.3), and CKD all significantly influenced the negative correlation. The interaction test showed that the effect of DI-GM on HUA varied by smoking history and the presence of diabetes (interaction *p* < 0.05).

**Table 4:** Subgroup analysis of the association between DI-GM and HUA.

**Subgroups**	**OR (95% CI) *p*-value**	***p* for interaction**
**Age**		
20–39	0.952 (0.916, 0.990) 0.015	0.402
40–59	0.996 (0.961, 1.032) 0.823	
≥60	0.957 (0.928, 0.987) < 0.005	
**Sex**		
Male	0.970 (0.944, 0.997) 0.027	0.936
Female	0.965 (0.937, 0.993) 0.016	
**Race**		
Mexican American	0.981 (0.921, 1.045) 0.551	0.112
Other Hispanic	0.924 (0.861, 0.992) 0.029	
Non-Hispanic white	0.978 (0.950, 1.006) 0.117	
Non-Hispanic black	0.991 (0.951, 1.034) 0.690	
Other race	0.922 (0.870, 0.979) 0.007	
**Education**		
Less than high school	0.999 (0.955, 1.045) 0.971	0.128
High school or equivalent	0.975 (0.937, 1.016) 0.227	
College or above	0.956 (0.932, 0.982) < 0.001	
**Marital status**		
Married and a partner	0.972 (0.947, 0.997) 0.029	0.703
Never married	0.980 (0.933, 1.029) 0.420	
Widowed, divorced, or separated	0.952 (0.914, 0.990) 0.015	
**Poverty to income ratio**		
< 1.3	0.994 (0.958, 1.032) 0.757	0.081
1.3–3.5	0.963 (0.934, 0.994) 0.020	
>3.5	0.950 (0.918, 0.984) 0.004	
**Smoking status**		
Never smoker	0.948 (0.923, 0.974) < 0.001	0.044
Current smoker	1.007 (0.960, 1.056) 0.787	
Former smoker	0.983 (0.948, 1.020) 0.363	
**Drinking status**		
Never drinker	0.957 (0.930, 0.984) 0.002	0.210
Current drinker	0.968 (0.936, 1.001) 0.058	
Former drinker	1.009 (0.961, 1.059) 0.716	
**Body mass index**		
Under weight (< 18.5)	0.899 (0.655, 1.233) 0.508	0.265
Normal (18.5–25)	0.948 (0.903, 0.995) 0.031	
Overweight (25–30)	0.944 (0.912, 0.978) 0.001	
Obese (30 or greater)	0.982 (0.956, 1.009) 0.190	
**Hypertension**		
No	0.968 (0.939, 0.997) 0.033	0.639
Yes	0.963 (0.939, 0.989) < 0.005	
**Diabetes**		
No	0.961 (0.939, 0.983) < 0.001	0.047
Yes	0.995 (0.956, 1.035) 0.795	
**Hyperlipidemia**		
No	0.950 (0.909, 0.993) 0.024	0.790
Yes	0.961 (0.940, 0.982) 0.002	
**CKD**		
No	0.968 (0.946, 0.991) 0.005	0.777
Yes	0.947 (0.911, 0.986) 0.007	

Stratified analysis based on model 3 was performed. In each case, the model was not adjusted for the stratification variable itself.

**Figure 3:**
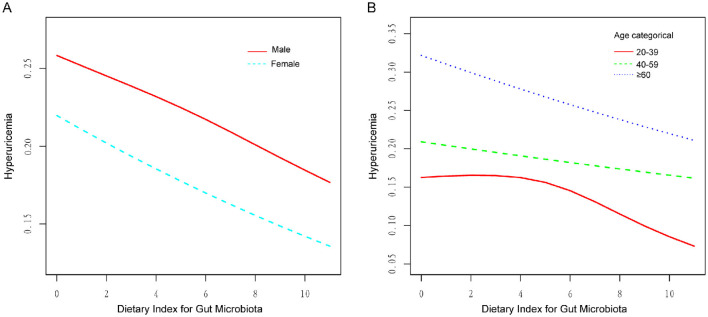
RCS of the association between DI-GM and HUA in different genders and ages. **(A)** RCS subgroup analysis by gender, showing a negative linear correlation in both groups. **(B)** RCS subgroup analysis by age, revealing a non-linear relationship in the 20–39 age group.

The RCS subgroup analysis showed that the DI-GM score had a consistent linear negative correlation with the risk of HUA in both genders (Figure 3A) and in individuals over 40 years old (Figure 3B). In the 20–39 age group, a non-linear relationship was observed (Figure 3B). When DI-GM < 4, there was no significant difference in the risk of HUA (OR = 1.016, 95% CI = 0.934–1.105, *p* = 0.709). However, when DI-GM ≥4, the risk of HUA decreased (OR = 0.897, 95% CI = 0.844–0.954, *p* < 0.005). The log-likelihood ratio test was 0.043.

## 4 Discussion

This research represents the initial exploration of the correlation between DI-GM scores and HUA, utilizing data from NHANES. The results indicate a notable inverse linear correlation between DI-GM scores and serum UA levels, implying that more favorable dietary habits could potentially lower the risk of HUA through the modulation of the GM. The findings are consistent with earlier studies that highlight the essential function of dietary composition in the regulation of UA metabolism (29). This study extends the application of DI-GM to hyperuricemia, reinforcing its utility in assessing diet quality in metabolic research. Notably, DI-GM scores showed a consistent linear negative correlation with the risk of HUA in both genders and in individuals over 40. In contrast, a non-linear relationship was observed in the 20–39 age group, with a negative association at DI-GM scores ≥4.

Dietary components are closely linked to the synthesis and excretion of UA. Numerous studies have shown that this relationship is mediated by the regulation of the GM (22, 30, 31). For instance, beneficial components in DI-GM, such as dietary fiber (32), fermented dairy products (33), coffee, and green tea (34), can alleviate HUA. On the other hand, unfavorable dietary components such as red meat and high-fat foods have been linked to elevated UA levels (8, 35). Dietary fiber, an essential part of a balanced diet, plays a crucial role in regulating the GM. Numerous studies have shown that fiber intake helps manage serum UA levels (13, 36). Research suggests (37, 38) that dietary fiber fosters the growth of beneficial bacteria, which, in turn, reduce UA synthesis by inhibiting key enzymes like xanthine oxidase in the liver (39, 40). Additionally, dietary fiber enhances intestinal motility, reducing purine absorption, which further supports its beneficial effects on HUA (41).

Short-chain fatty acids (SCFAs) are the main metabolites generated through the fermentation of dietary fiber by the GM. Short-chain fatty acids serve as a source of energy for the intestinal epithelial cells, aiding in the excretion of UA and lowering the risk of HUA (42). Tea and its bioactive compounds, especially polyphenols, contribute to the management of HUA. Polyphenols inhibit xanthine oxidase and regulate UA transporters, indicating that these compounds could act as molecular targets for the anti-hyperuricemia effects of tea (43). Polyphenols, found in high concentrations in various plant-based foods such as tea (44), have demonstrated the ability to enhance the population of beneficial bacteria including *Bifidobacterium, Lactobacillus, Bacteroides*, and *Prevotella*. Concurrently, they reduce the relative abundance of Proteobacteria and alter the Firmicutes-to-*Bacteroides* ratio, thereby acting as oral prebiotics (45).

Conversely, specific detrimental elements of the DI-GM, are significant characteristics of the Western dietary pattern, which has been linked to a heightened risk of HUA (46). Earlier research indicates that the negative consequences of these dietary elements on hyperuricemia are influenced by their interaction with the GM (21). For example, *Lactobacillus plantarum* has demonstrated the ability to mitigate the increase in UA levels prompted by high-fat diets (47). In a 10-week observational crossover study, 20 healthy adults participated in the consumption of two isocaloric diets: one abundant in whole grains and fiber, and the other predominantly consisting of red meat. The consumption of red meat induced notable alterations in the gut microbiota, especially regarding the prevalence of Firmicutes, and was associated with elevated serum levels of UA and creatinine (48).

The stratified analysis conducted in this study indicated that the association between DI-GM scores and HUA differed across age groups. In individuals over the age of 40, a steady negative linear correlation was noted between DI-GM scores and the risk of HUA. Conversely, in those aged 20–39, the relationship between DI-GM scores and HUA exhibited a non-linear pattern. Low DI-GM scores did not significantly affect UA levels, which may be due to age-related metabolic changes, such as decreased glomerular density and reduced UA excretion capacity (49). A healthy dietary pattern may play a more significant protective role in this population by improving GM and enhancing metabolic function.

Given that increasing DI-GM scores can reduce the risk of HUA, we suggest that dietary patterns should be modified to increase DI-GM scores rather than simply restricting high-purine foods. This approach may help prevent HUA. For instance, seaweed, dried laver, squid, and other marine-based foods, previously considered rich in purines and should be consumed cautiously by individuals with HUA, may be included in the diet as part of an overall healthy dietary pattern. Other studies have found negative correlations between DI-GM scores and the prevalence of stroke (24), diabetes (25), and metabolic dysfunction-associated fatty liver disease (26).

Although the DI-GM was originally developed to evaluate the microbiota-related quality of diet, the findings of this study suggest that it may also be useful in guiding dietary strategies for the prevention of HUA. Unlike traditional dietary recommendations that emphasize purine restriction, the DI-GM captures a wider range of dietary components, including fiber, polyphenols, and fermented foods, which are known to influence gut microbiota composition and UA metabolism. The inverse association observed between DI-GM and HUA supports its potential role in identifying dietary patterns associated with lower risk. While the cross-sectional design of this study limits causal interpretation, the results highlight the importance of incorporating microbiota-related dietary quality into future research and nutritional approaches targeting HUA.

This study thoroughly examines the interplay between DI-GM and the occurrence of HUA, providing fresh insights into the intricate relationships among nutrition, microbial communities, and UA metabolism. In light of these observations, it is important to acknowledge the various constraints present. First, the cross-sectional design of this study precludes causal inference, as it does not allow for the assessment of temporal sequence or directionality between DI-GM scores and hyperuricemia. Future longitudinal cohort studies and randomized controlled trials are warranted to verify whether improvements in DI-GM can directly reduce the risk or progression of HUA. Second, although the DI-GM was constructed based on established literature linking dietary components to gut microbiota characteristics, it has not yet been directly validated against microbial composition or functional profiles derived from sequencing data. Existing associations between DI-GM and microbiota-related diseases provide only indirect support for its construct validity. Further microbiome-based validation is needed to enhance the credibility of this index. Third, the study data is derived from the U.S. population, which may limit the generalizability of the findings to other populations.

While causal inferences cannot be drawn due to the cross-sectional design, our findings suggest that the DI-GM serves as an exploratory tool to characterize gut microbiota-related dietary patterns associated with UA metabolism. The score is derived from standard 24-h dietary recall data, making it applicable in large-scale nutritional epidemiology. The observed age-related differences, including a stable inverse association in adults aged 40 years and older and a non-linear trend among younger adults, highlight the importance of considering population heterogeneity in diet–microbiota–host interactions. These patterns provide a basis for refining stratification strategies in future observational studies. To further support the present findings, future studies should aim to replicate these associations in independent, population-based datasets with comparable dietary and biochemical measures. Such validation would help assess the external validity of the DI-GM across diverse demographic and nutritional contexts. Additionally, applying the DI-GM in clinically relevant subgroups, such as individuals with obesity, insulin resistance, or impaired renal function, may help determine whether its association with hyperuricemia differs by metabolic condition. Although direct microbiome data were not available in this study, linking the DI-GM to validated microbiota-related biomarkers or evaluating it in datasets that include both dietary and microbial data could provide additional insight into its biological relevance.

## 5 Conclusion

The present study identified a significant inverse association between DI-GM scores and hyperuricemia. These findings suggest a potential relationship between microbiota-related dietary patterns and uric acid metabolism that warrants further investigation.

## Data Availability

The original contributions presented in the study are included in the article/supplementary material, further inquiries can be directed to the corresponding author.
